# Spectral Optimization of Programmable LED Illumination for Smartphone-Based Colorimetric Biosensor Readout

**DOI:** 10.3390/bios16100559

**Published:** 2026-10-03

**Authors:** Elena Serea, Codrin Donciu, Marinel Costel Temneanu

**Affiliations:** Faculty of Electrical Engineering, “Gheorghe Asachi” Technical University of Iași, 700050 Iași, Romania; codrin.donciu@academic.tuiasi.ro (C.D.); marinel-costel.temneanu@academic.tuiasi.ro (M.C.T.)

**Keywords:** smartphone colorimetry, colorimetric biosensors, programmable LED illumination, spectral power distribution, CIEDE2000, spectral optimization, image saturation, colorimetric readout

## Abstract

Smartphone-based colorimetric biosensor readout is strongly influenced by illumination, yet lighting conditions are often characterized mainly by illuminance rather than by their spectral composition. This study investigates the spectral, photometric, colorimetric, and energetic effects of programmable LED illumination on smartphone colorimetric readout under controlled acquisition conditions. Twenty lighting scenarios were generated using three independently controlled color-capable LED luminaires and characterized by spectral irradiance, illuminance, chromaticity, color rendering, and electrical power. A Samsung Galaxy S20 FE was used to acquire JPEG and RAW images of a standardized 24-patch ColorChecker target used as a controlled colorimetric reference for evaluating the optical readout methodology, and colorimetric deviation was quantified using CIEDE2000. Mean ΔE_00_ ranged from 11.73 to 42.33 across the investigated scenarios. Two conditions differing by less than 1% in illuminance produced a 37% difference in mean colorimetric error, demonstrating that illuminance alone does not determine readout accuracy. An illuminance-only regression model yielded R^2^ = 0.819, whereas inclusion of a relative spectral descriptor increased R^2^ to 0.919. High-output conditions also revealed substantial JPEG clipping despite negligible RAW saturation. These results show that reliable smartphone colorimetric readout requires joint consideration of illumination intensity, spectral composition, electrical demand and image-saturation constraints.

## 1. Introduction

Smartphone-based optical sensing has become an important platform for portable and point-of-care chemical and biological analysis, combining integrated imaging, computation, connectivity, and increasingly sophisticated data-processing capabilities [1,2,3,4,5,6,7,8,9]. Camera-based colorimetric approaches are particularly attractive because chromogenic responses from lateral-flow assays, microfluidic devices, paper-based sensors and other analytical formats can be converted directly into quantitative image data [1,2,3,4,5,6,10]. Recent developments continue to demonstrate the relevance of smartphone imaging for decentralized biosensing, including controlled-lighting systems for quantitative biomarker analysis [11]. However, the RGB response recorded by a smartphone camera is not determined solely by the intrinsic color of the sensing region. For a camera channel *i*, the recorded signal can be represented conceptually as:

(1) $$C_{i}\propto \int E\left(\lambda \right)R\left(\lambda \right)S_{i}\left(\lambda \right)\mathrm{d\lambda}$$

where *E*(*λ*) is the spectral power distribution of the incident illumination, *R*(*λ*) is the spectral reflectance of the target, and *S_i_*(*λ*) is the spectral sensitivity of the corresponding camera channel. Thus, variations in the illumination spectrum can modify the recorded RGB response even when the target itself remains unchanged [12,13,14]. This effect is further complicated by device-dependent camera spectral sensitivities and by the internal processing applied during generation of smartphone JPEG images [12,14,15]. The recent literature further confirms the growing use of smartphone-based optical and colorimetric analysis in biomedical and analytical applications [16,17]. Experimental studies have also shown that smartphone color measurements depend on color-space selection, reference-target calibration, device characteristics, and illumination conditions [18,19,20,21].

Illumination is therefore a measurement parameter of the complete optical readout chain rather than merely a means of increasing scene brightness. Controlled illumination and fixed acquisition geometries are commonly employed to reduce variability in smartphone colorimetry and biosensing [1,2,10,11,15]. Nevertheless, illuminance alone cannot uniquely describe the spectral stimulus incident on the camera because it is photometrically weighted according to the human visual sensitivity function V(λ). Consequently, two lighting conditions having similar illuminance may produce different camera responses when their spectral power distributions differ. The importance of source spectral composition has been demonstrated in RGB-camera measurements, where optimization of the illumination spectrum can improve spectral-reconstruction performance [13]. Programmable multichannel LED sources provide a practical means of modifying both intensity and spectral composition, and spectral optimization has also been investigated in lighting systems with respect to color quality and energetic performance [22,23]. For smartphone-based quantitative imaging, however, increasing optical output introduces an additional constraint because the processed image may reach saturation even when useful sensor-level information remains available in the RAW data [2,15].

The present study investigates programmable LED illumination as an actively controlled measurement variable for smartphone-based colorimetric biosensor readout. Twenty illumination scenarios generated by three independently controlled color-capable LED sources were experimentally characterized in terms of spectral irradiance, illuminance, chromaticity, color rendering, and electrical power. A standardized 24-patch ColorChecker target was used as a controlled colorimetric reference, and smartphone readout performance was quantified using CIEDE2000 color differences. The analysis was designed to separate the contribution of illumination level from that of spectral composition, examine color-dependent responses, evaluate experimental repeatability, and assess JPEG and RAW saturation behavior. To address the resulting measurement challenge, the study evaluates programmable illumination as an active component of the smartphone colorimetric measurement chain, rather than as a fixed lighting condition, and introduces a joint spectral-colorimetric-energetic framework that additionally accounts for image-saturation constraints.

## 2. Materials and Methods

### 2.1. Experimental Setup and Lighting Scenarios

**Figure 1:**
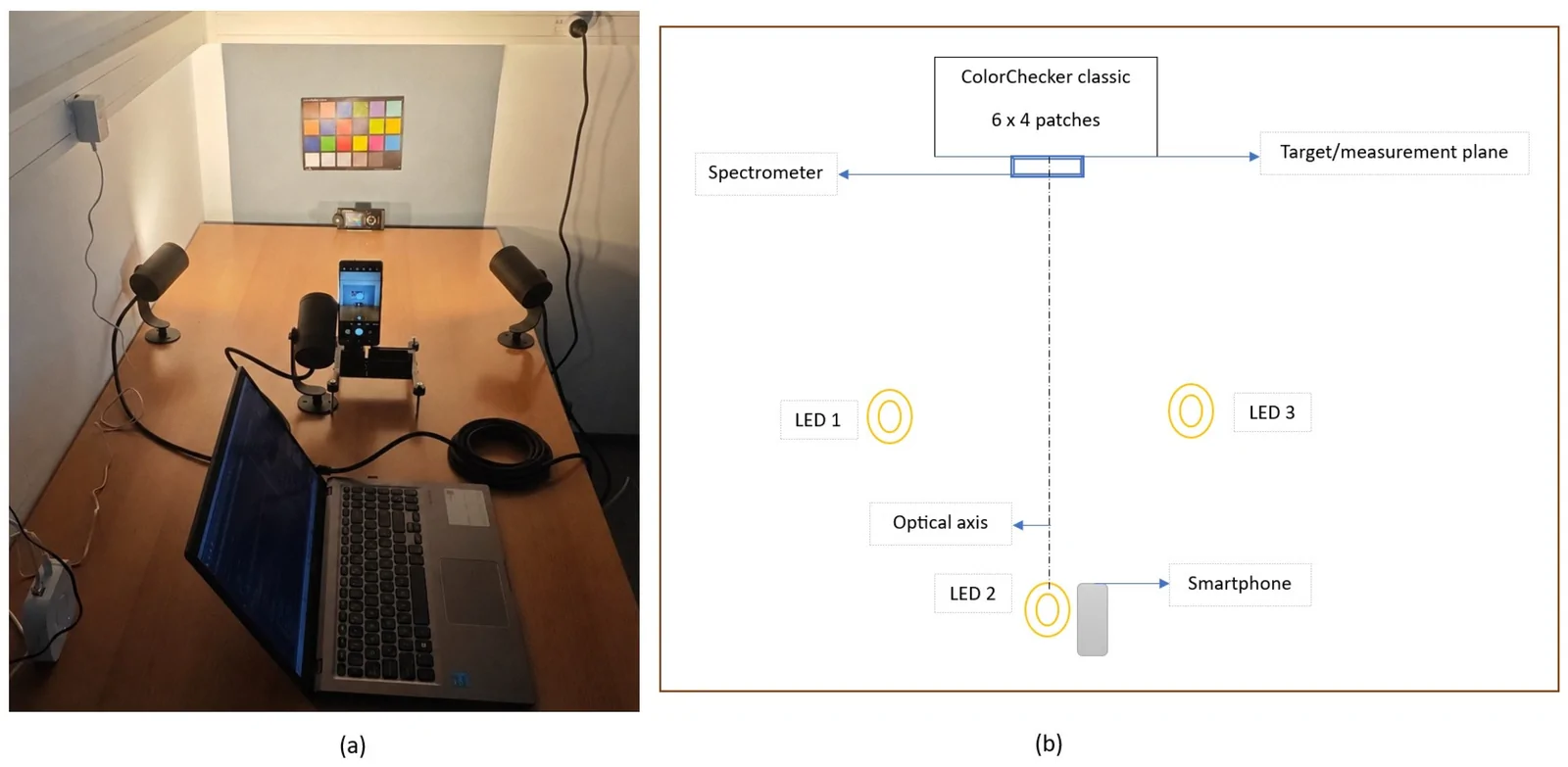
Experimental setup used for the evaluation of programmable LED illumination for smartphone-based colorimetric readout: (**a**) photograph of the measurement arrangement; (**b**) simplified top-view schematic showing the relative positions of the three LED luminaires, the ColorChecker target, the spectrometer at the target/measurement plane and the smartphone camera.

The experimental platform was designed to investigate the influence of programmable LED illumination on smartphone-based colorimetric readout under controlled acquisition conditions. The setup (Figure 1) included 3 Philips Hue Lily color-capable LED luminaires and a Philips Hue Bridge (Signify The Netherlands B.V. Eindhoven) for wireless control, a Calibrite ColorChecker Classic 24-patch target (Calibrite LLC, Wilmington, DE, USA), a MAVO-SPEC BASE spectrometer (GOSSEN Foto- und Lichtmesstechnik GmbH, Nuremberg, Germany) for optical characterization, a Tapo P115 smart plug (TP-Link Systems Inc., Irvine, CA, USA) for electrical power measurements and a Samsung Galaxy S20 FE (SM-G780G) smartphone (Samsung Electronics Co., Ltd., Suwon, Republic of Korea) for image acquisition.

The ColorChecker target was mounted vertically on a matte gray background. The three luminaires were positioned in front of the target so as to provide combined illumination over the full chart area. The smartphone and spectrometer were both aligned with the target plane, and the relative positions of all components were kept unchanged throughout the measurement campaign.

To improve the reproducibility of the experimental arrangement, the main geometric parameters of the setup were measured directly and are summarized in Table 1. Distances were referenced to the center of the ColorChecker target, while heights correspond to the optical centers of the respective components. Azimuth was defined relative to the normal to the ColorChecker plane, with negative and positive values denoting positions on opposite sides of the optical axis. The vertical tilt angle of each LED luminaire was measured relative to the horizontal plane. The corresponding LED 1-target-LED 2 and LED 2-target-LED 3 angular separations were 30° in both cases. All component positions and orientations were kept unchanged throughout the experimental measurements.

Illumination conditions were generated by software control of the LEDs using Python and phue library, with hue, saturation and brightness settings specified for each source. S01–S20 lighting scenarios were defined to provide systematic variations in illumination level and spectral composition, as summarized in Table 2. Scenario S20 repeating S14 was included for repeatability assessment. The nominal source settings were used only to define the commanded lighting states.

To assess image-acquisition repeatability, an additional acquisition series was performed for all 20 lighting scenarios. Three separate smartphone image acquisitions were obtained for each scenario under unchanged camera settings and nominal illumination conditions, resulting in 60 additional acquisitions, each simultaneously stored in JPEG and DNG formats. They were processed independently using the same ROI-based colorimetric workflow as the primary dataset. Repeatability was evaluated from the variability of the scenario-level mean ΔE_00_ values across the three acquisitions. These additional acquisitions were used specifically for repeatability assessment and were not substituted for the primary image dataset paired with the spectroradiometric characterization.

### 2.2. Optical and Electrical Characterization

For each lighting scenario, the combined illumination produced by the 3 luminaires was characterized at the ColorChecker target plane using a MAVO-SPEC BASE spectrometer. The measured parameters included illuminance E [lx], irradiance Ee [W/m^2^], correlated color temperature (CCT), general color rendering index CRI (Ra) and CIE 1931 chromaticity coordinates x and y. Spectral irradiance distributions were recorded from 380 to 780 nm at 5 nm intervals.

To compare spectral shape independently of absolute output level, each measured spectral irradiance distribution *SPD*(*λ*) was normalized to its maximum value:

(2) $${\mathrm{SPD}}_{N}\left(\lambda \right)=\frac{\mathrm{SPD}(\lambda )}{\max_{\lambda }[\mathrm{SPD}\left(\lambda \right)]}$$

Electrical power consumption was measured for each scenario using a Tapo P115 smart plug. Three consecutive power readings were recorded, and the results are reported as mean ± standard deviation. In addition, the ratio between target-plane illuminance and electrical power, E/P [lx/W], was calculated. This quantity was used as an illuminance-to-power indicator for comparing the experimental lighting conditions and should not be interpreted as the luminous efficacy of the luminaires.

### 2.3. Smartphone Image Acquisition and Colorimetric Analysis

Images were acquired using a Samsung Galaxy S20 FE (SM-G780G) smartphone with the main 1× camera in Pro mode. The acquisition geometry was kept unchanged for all lighting scenarios. Camera settings were fixed at ISO 100 and an exposure time of 1/125 s, with a constant white-balance setting throughout the experiment. Autofocus was used to ensure consistent chart sharpness. For each lighting scenario, one image acquisition was performed and simultaneously stored in JPEG and DNG (RAW) formats. The JPEG and DNG files therefore represent two formats of the same acquisition and were not treated as independent replicates.

Colorimetric analysis was performed on the JPEG images using a custom Python-based image-processing workflow. The ColorChecker chart was localized in each image, its four corners were identified, and the standard 6 × 4 patch geometry was mapped to the detected target. Central regions of interest (ROIs) were defined within each of the 24 patches to minimize the influence of patch borders and residual geometric distortions. RGB values used for the colorimetric calculations were extracted from the original image pixels. The JPEG images were interpreted in the sRGB color space. Mean patch RGB values were linearized and converted to CIE XYZ under D65, chromatically adapted to D50, and subsequently transformed to CIELAB coordinates. The resulting values were compared with the reference CIELAB values of the Calibrite ColorChecker Classic under the D50/2° reference condition. Colorimetric deviation for each patch was quantified using the CIEDE2000 color-difference metric, ΔE_00_. For each lighting scenario, the mean, standard deviation, median, and maximum ΔE_00_ values across the 24 patches were calculated. The standard deviation reported for ΔE_00_ therefore represents variability among the 24 ColorChecker patches and not image-acquisition repeatability.

### 2.4. Spectral Descriptors, Repeatability and Statistical Analysis

To investigate the influence of spectral composition independently of illumination level, a relative spectral descriptor, *G_rel_*, was calculated as the fraction of the measured spectral irradiance contained within the 500–600 nm band relative to the total spectral irradiance between 380 and 780 nm:

(3) $$G_{\mathrm{rel}}=\frac{\int_{500}^{600}\mathrm{SPD}\left(\lambda \right)\mathrm{d\lambda}}{\int_{380}^{780}\mathrm{SPD}\left(\lambda \right)\mathrm{d\lambda}}$$

The 500–600 nm interval was selected as a broad spectral region encompassing the region around the peak of the photopic sensitivity function. Its relationship with colorimetric performance was subsequently evaluated empirically.

The relationship between mean ΔE_00_ and target-plane illuminance was first evaluated using linear regression against the natural logarithm of illuminance, ln(E). For each lighting scenario, the residual colorimetric error was calculated as the difference between the measured mean ΔE_00_ and the value predicted by the illuminance-only model. The association between the residuals and *G_rel_* was evaluated using Pearson’s correlation coefficient. A second multiple linear regression model incorporating both ln(E) and *G_rel_* was subsequently fitted, and the two models were compared using R^2^ and adjusted R^2^. Repeatability of the experimental conditions was evaluated by comparing S14 with its repeated condition S20. Spectral-shape agreement was quantified using cosine similarity and the root mean square error (RMSE) between the corresponding normalized spectral irradiance distributions. Repeatability was additionally evaluated using the measured optical parameters and the resulting mean ΔE_00_ values.

JPEG and RAW saturation behavior was evaluated within the same 24 central ColorChecker regions of interest (ROIs) used for the colorimetric analysis. For JPEG images, a pixel was classified as clipped when at least one 8-bit RGB channel reached a value of 254 or higher. For DNG data, clipping was identified when at least one reconstructed sensor-channel value reached the DNG white-level threshold (WhiteLevel −1). Clipping percentages were calculated separately for each patch and image format. Mean values represent the average clipped-pixel fraction across the 24 patches, whereas maximum values correspond to the most affected individual patch.

## 3. Results

### 3.1. Spectral, Photometric, Radiometric and Colorimetric Characterization of the Lighting Scenarios

Table 3 summarizes the photometric, radiometric, colorimetric, and electrical characteristics measured for the 20 experimental lighting scenarios. The target-plane illuminance ranged from 65.5 lx to 1355.25 lx, while the corresponding irradiance ranged from 0.223 W/m^2^ to 4.414 W/m^2^. The measured correlated color temperature varied substantially among the scenarios, from 2576 K to 5541 K, reflecting the intended transition from warm to cool-dominant illumination. The general color rendering index remained relatively high across the investigated conditions, with CRI (Ra) values between 88.1 and 95.1. The CIE 1931 chromaticity coordinates reflected the expected chromaticity shifts among the warm-, neutral-, cool-, and mixed-light conditions.

**Figure 2:**
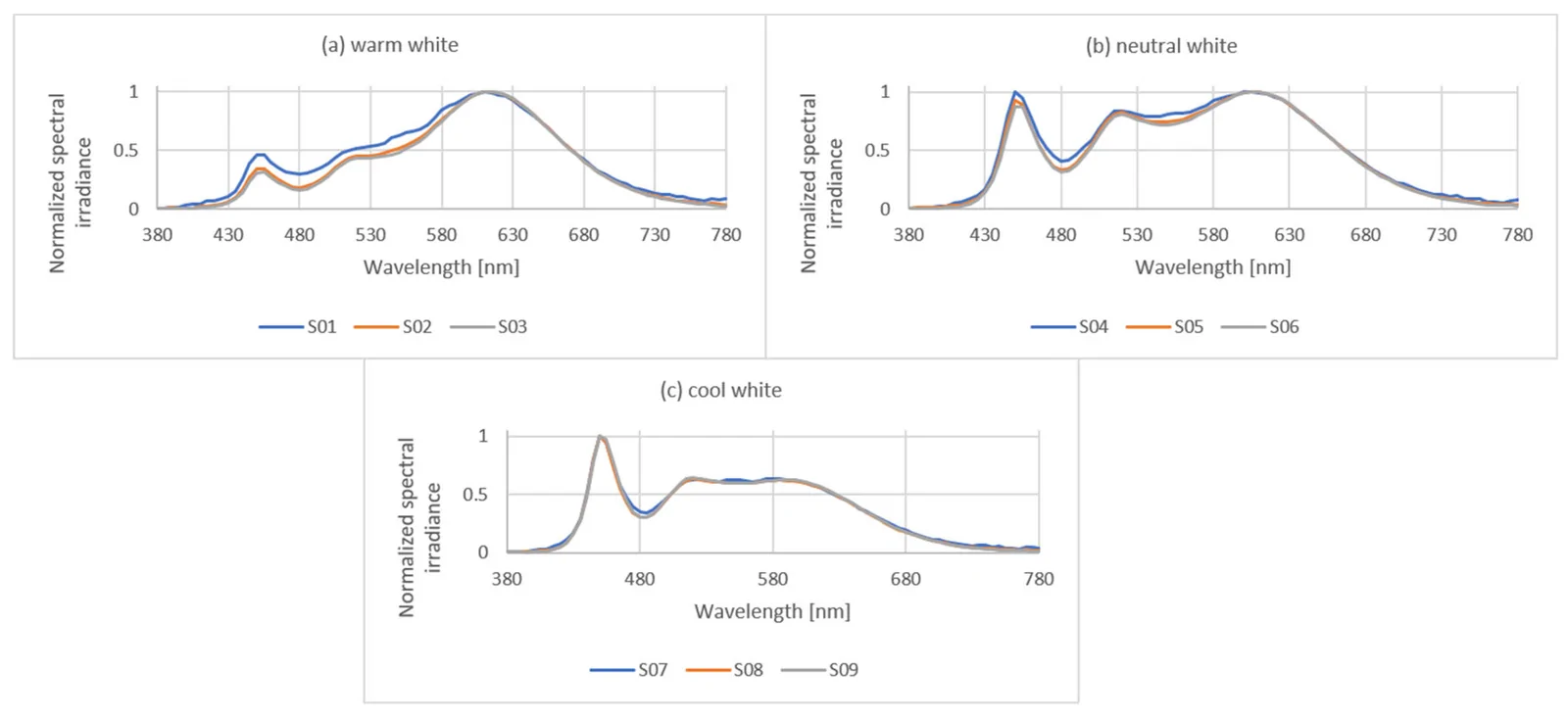
Normalized spectral irradiance distributions of the white-light scenarios at different brightness levels: (**a**) warm white, S01–S03; (**b**) neutral white, S04–S06; and (**c**) cool white, S07–S09.

Figure 2 compares the normalized spectral irradiance distributions of the warm-, neutral-, and cool-white scenarios at different brightness levels. The warm-white conditions (S01–S03) exhibited a broad dominant contribution in the longer-wavelength region, with a maximum located approximately in the 600–620 nm range. Although the overall spectral shape remained similar across the three brightness levels, the lowest-brightness condition, S01, showed a relatively stronger short-wavelength contribution around 450 nm than S02 and S03. This indicates that brightness variation did not result exclusively in a proportional scaling of spectral amplitude, but also introduced small changes in the normalized spectral profile. A similar behavior was observed for the neutral-white scenarios (S04–S06). Their spectra were characterized by a pronounced blue peak near 450 nm combined with a broad longer-wavelength emission band. The three normalized curves remained closely grouped, although S04 again exhibited a slightly higher relative blue contribution compared with the higher-brightness conditions. These differences suggest a modest brightness-dependent modification of the spectral balance. In contrast, the cool-white scenarios (S07–S09) showed substantially greater spectral-shape stability across brightness levels. All three spectra displayed a dominant short-wavelength peak near 450 nm and a broader secondary contribution extending approximately from 500 to 620 nm. The close overlap of the normalized curves indicates that, for the cool-white setting, brightness modulation predominantly affected the absolute output level while preserving the relative spectral distribution.

**Figure 3:**
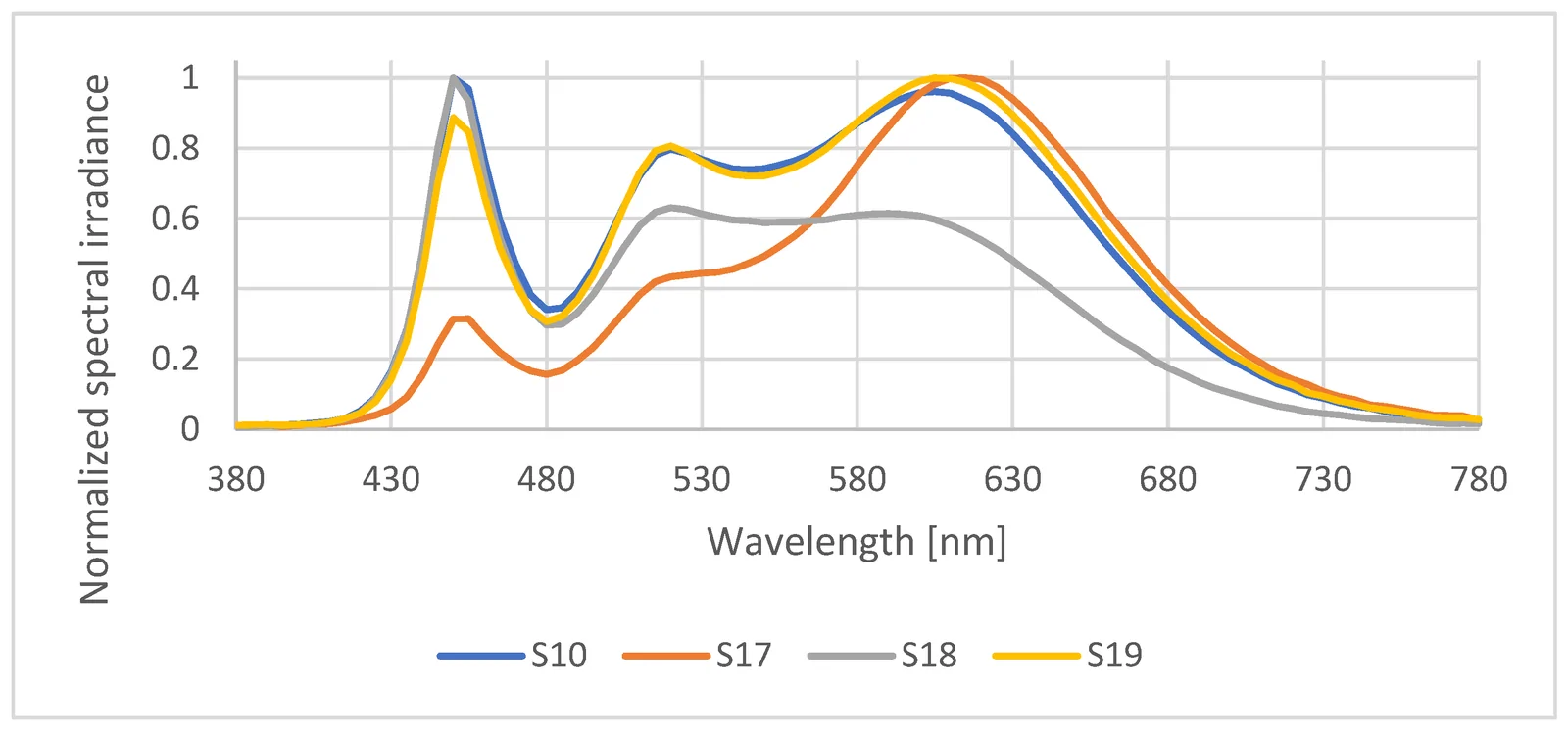
Normalized spectral irradiance distributions of the mixed-white scenario S10 and the unequal-intensity warm-, cool-, and neutral-white scenarios S17–S19.

Figure 3 presents the normalized spectral irradiance distributions of the mixed-white scenario S10 and the unequal-intensity white-light scenarios S17–S19. In S17, S18, and S19, the three luminaires were operated using the same brightness distribution of 25%, 50%, and 75%, while the chromatic setting was maintained as warm white, cool white, and neutral white, respectively.

The warm-white condition S17 exhibited the lowest relative contribution in the short-wavelength region and the strongest relative contribution at longer wavelengths. Conversely, S18 showed a pronounced blue peak and a substantially reduced long-wavelength contribution, consistent with its cool-white character. The neutral-white condition S19 occupied an intermediate spectral position between S17 and S18.

The mixed-white condition S10, obtained by simultaneously combining warm-, neutral-, and cool-white sources at equal brightness, produced an intermediate spectral profile that closely approached, but did not fully reproduce, the neutral-white condition S19.

**Figure 4:**
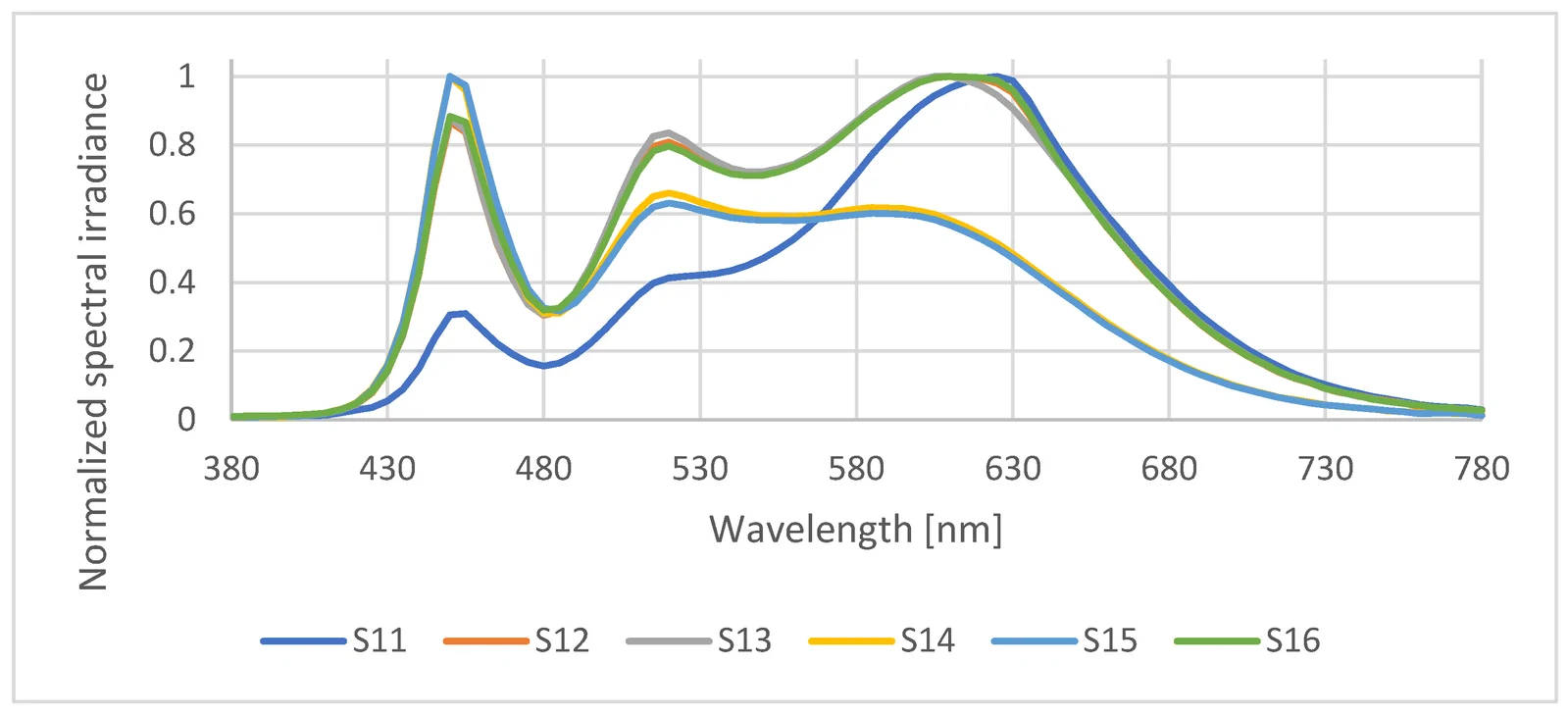
Normalized spectral irradiance distributions of the color-accented lighting scenarios S11–S16. Each scenario combined one color-accented LED source with two white LED sources, resulting in hybrid spectral profiles rather than pure monochromatic emissions.

Figure 4 shows the normalized spectral irradiance distributions of the color-accented scenarios S11–S16. In contrast to monochromatic reference sources, these spectra remained hybrid because each scenario combined one color-accented Hue source with two white sources. Consequently, all conditions preserved the characteristic short-wavelength LED peak near 450 nm together with a broader emission band extending across the mid- and long-wavelength regions.

Among the investigated scenarios, S11 exhibited the most pronounced long-wavelength dominance, consistent with its red-accented configuration. In this case, the relative contribution in the short-wavelength region was markedly reduced, while the spectral maximum shifted toward the 600–630 nm range. Scenarios S12 and S13 produced intermediate spectral profiles, with enhanced mid-spectral content compared with S11. By contrast, S14 and S15 showed stronger short-wavelength contributions, as expected for cyan- and blue-accented conditions. Scenario S16 combined an elevated short-wavelength contribution with a strong longer-wavelength component, yielding a more balanced dual-region spectral enhancement.

**Figure 5:**
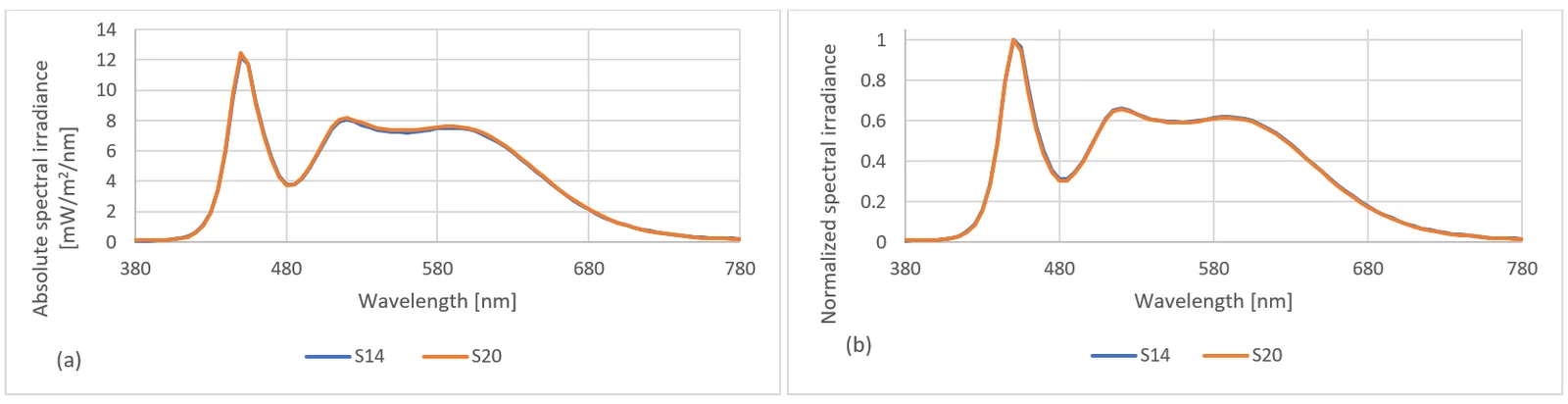
Spectral repeatability of the lighting setup evaluated by comparing scenario S14 with its repeated acquisition S20: (**a**) absolute spectral irradiance and (**b**) normalized spectral irradiance.

The repeatability of the lighting setup was assessed by repeating scenario S14 as S20. As shown in Figure 5a, the two absolute spectral irradiance distributions closely overlapped across the entire measured wavelength range. The integrated irradiance values were 1.680 W/m^2^ for S14 and 1.697 W/m^2^ for S20, corresponding to a difference of approximately 1.0%. After normalization, the spectral profiles remained nearly indistinguishable (Figure 5b), with a cosine similarity of 0.99995 and a normalized spectral RMSE of approximately 0.0055. These results indicate very good reproducibility of both the spectral shape and the emitted radiant level under repeated lighting conditions.

### 3.2. Colorimetric Performance of the Smartphone Readout

Table 4 summarizes the colorimetric performance obtained from the smartphone JPEG images under the 20 experimental lighting scenarios. For each scenario, the CIEDE2000 color difference (ΔE_00_) was calculated for all 24 ColorChecker patches, and the mean, standard deviation, median, and maximum values were determined. The mean ΔE_00_ varied substantially across the investigated lighting conditions, ranging from 11.73 for S09 to 42.33 for S01. The lowest median color difference was also obtained for S09 (9.73), whereas S01 exhibited the highest maximum deviation (71.57). These results demonstrate a strong dependence of the smartphone colorimetric response on the illumination scenario.

**Figure 6:**
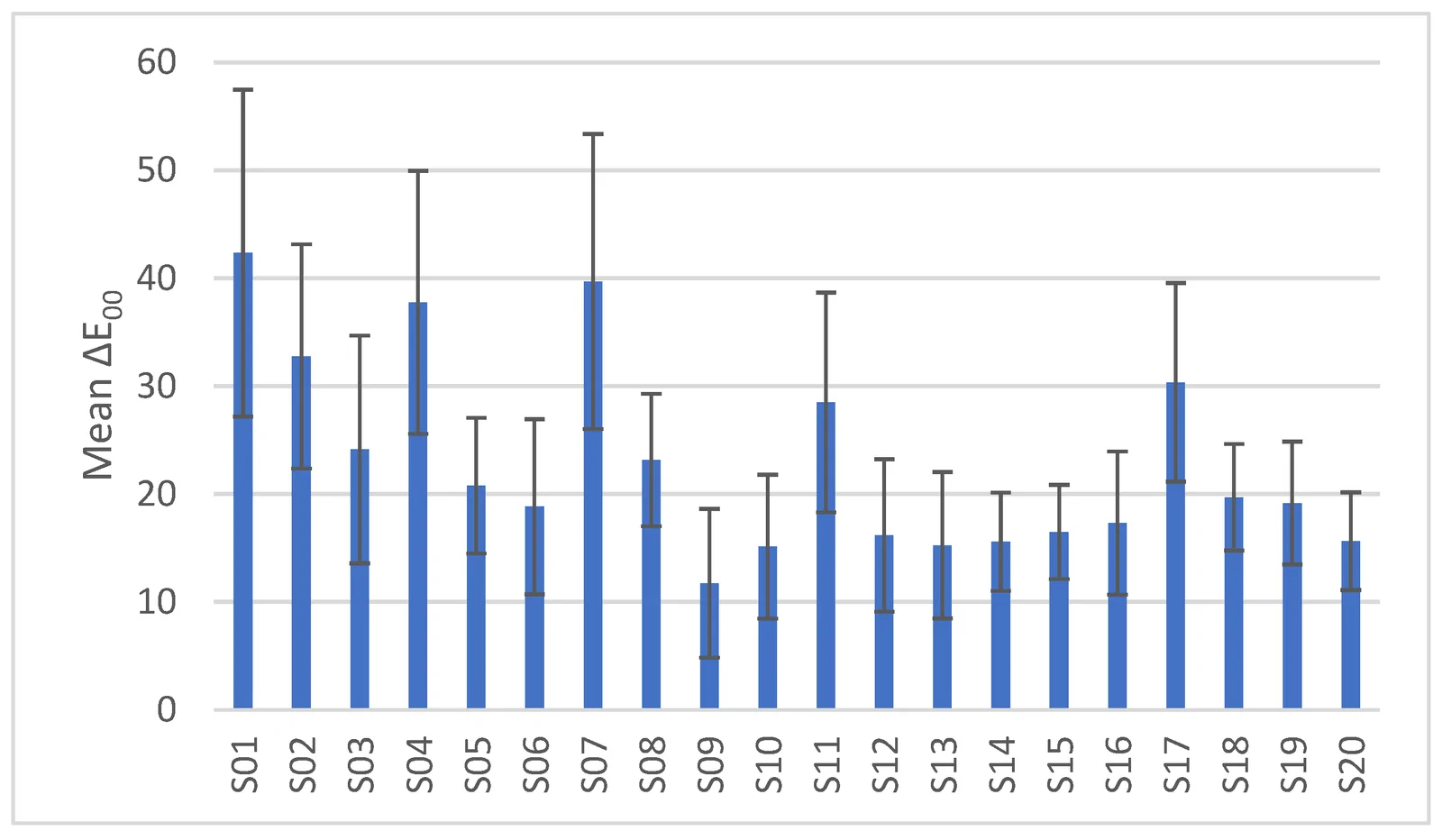
Mean CIEDE2000 color difference (ΔE_00_) obtained for the 20 experimental lighting scenarios. Error bars represent the standard deviation across the 24 ColorChecker patches.

To facilitate comparison among the lighting conditions, the mean ΔE_00_ values and their corresponding standard deviations across the 24 ColorChecker patches are presented graphically in Figure 6. It summarizes the mean CIEDE2000 color difference (ΔE_00_) obtained across the 24 ColorChecker patches for each lighting scenario. A clear reduction in colorimetric error was observed with increasing brightness within all three uniform white-light families. For warm-white illumination, the mean decreased from 42.33 in S01 to 32.76 in S02 and 24.13 in S03. A similar trend was observed for neutral white, with values decreasing from 37.76 (S04) to 20.78 (S05) and 18.83 (S06), and for cool white, from 39.70 (S07) to 23.16 (S08) and 11.73 (S09). These results indicate that, under fixed smartphone acquisition settings, increasing the illumination level generally improved the colorimetric readout. Illumination level alone did not determine colorimetric performance. The lowest mean error was obtained for S09 (ΔE_00_ = 11.73), although S09 was not the scenario with the highest measured illuminance. In comparison, S06 provided the highest target-plane illuminance (1355.25 lx) but resulted in a higher mean color difference of 18.83. This indicates that increasing illuminance does not necessarily lead to the lowest colorimetric error and suggests that the spectral composition of the incident illumination also plays an important role in smartphone-based colorimetric readout.

The mixed and color-accented scenarios S10–S16 generally produced intermediate mean values, ranging from 15.13 to 28.48. The repeated scenario S20 yielded a mean ΔE_00_ of 15.63, closely matching the value of 15.58 obtained for S14. This close agreement is consistent with the spectral repeatability previously observed for the same pair of scenarios.

The error bars in Figure 6 represent the standard deviation across the 24 ColorChecker patches and therefore reflect the dependence of the colorimetric error on patch color rather than image-acquisition repeatability.

**Figure 7:**
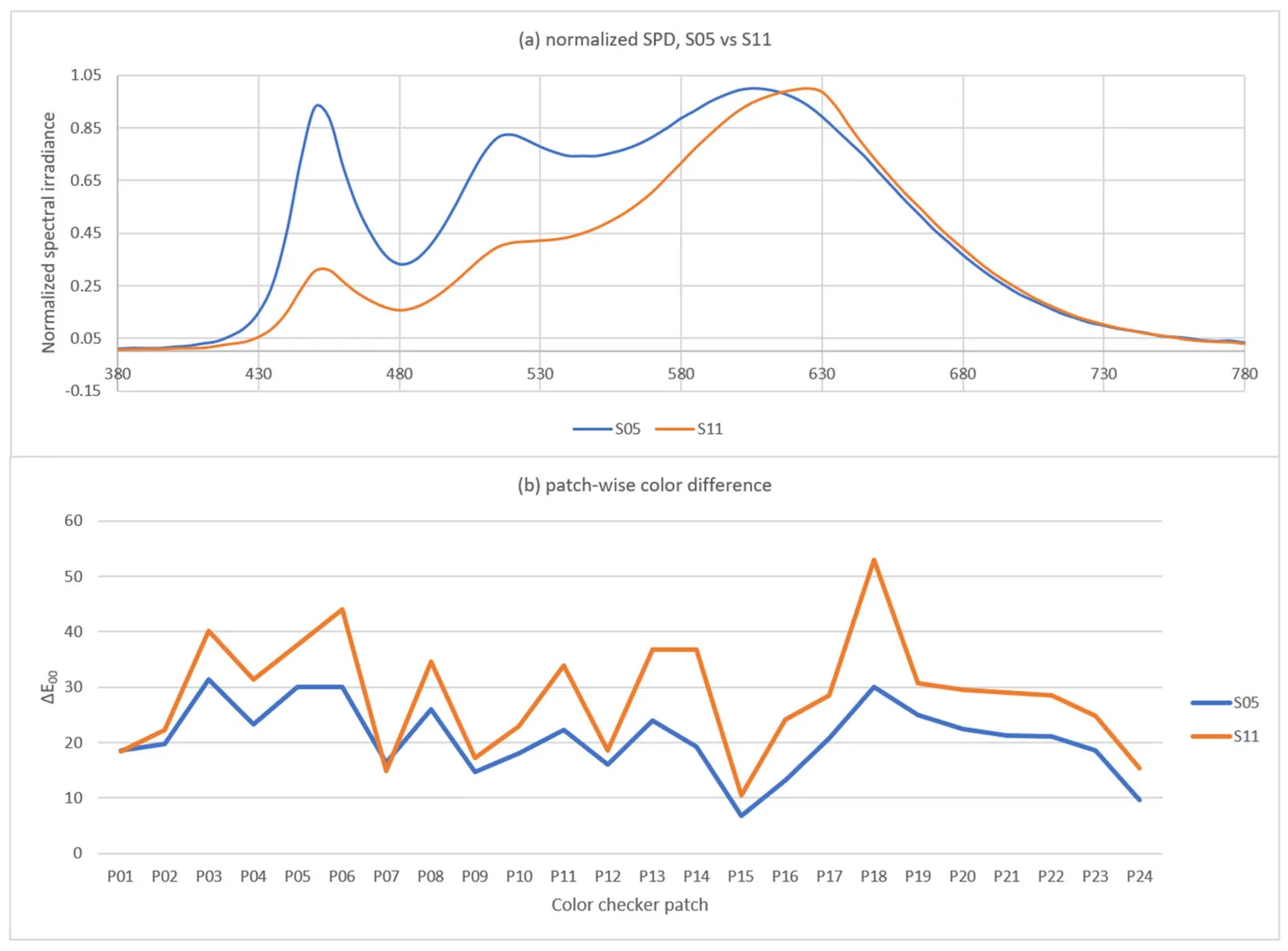
Comparison of two lighting scenarios with nearly identical target-plane illuminance but different spectral compositions: (**a**) normalized spectral irradiance distributions of S05 and S11; (**b**) CIEDE2000 color differences (ΔE_00_) obtained for the 24 ColorChecker patches.

To further examine the influence of spectral composition independently of illuminance, two scenarios with nearly identical target-plane illuminance were selected for direct comparison. Figure 7 compares S05 and S11, which provided illuminance levels of 354.33 lx and 357.15 lx, respectively, corresponding to a difference of less than 1%. Despite this close photometric similarity, their normalized spectral irradiance distributions were clearly different (Figure 7a), and the resulting patch-wise colorimetric errors also differed substantially (Figure 7b).

Despite their nearly identical target-plane illuminance (354.33 lx for S05 and 357.15 lx for S11), the two scenarios produced markedly different spectral distributions and colorimetric responses. The mean ΔE_00_ increased from 20.78 for S05 to 28.48 for S11, corresponding to an increase of approximately 37%. The effect was strongly patch-dependent, with particularly large differences observed for several blue-green patches, including Cyan (P18), for which ΔE_00_ increased from approximately 30.0 to 52.9.

**Figure 8:**
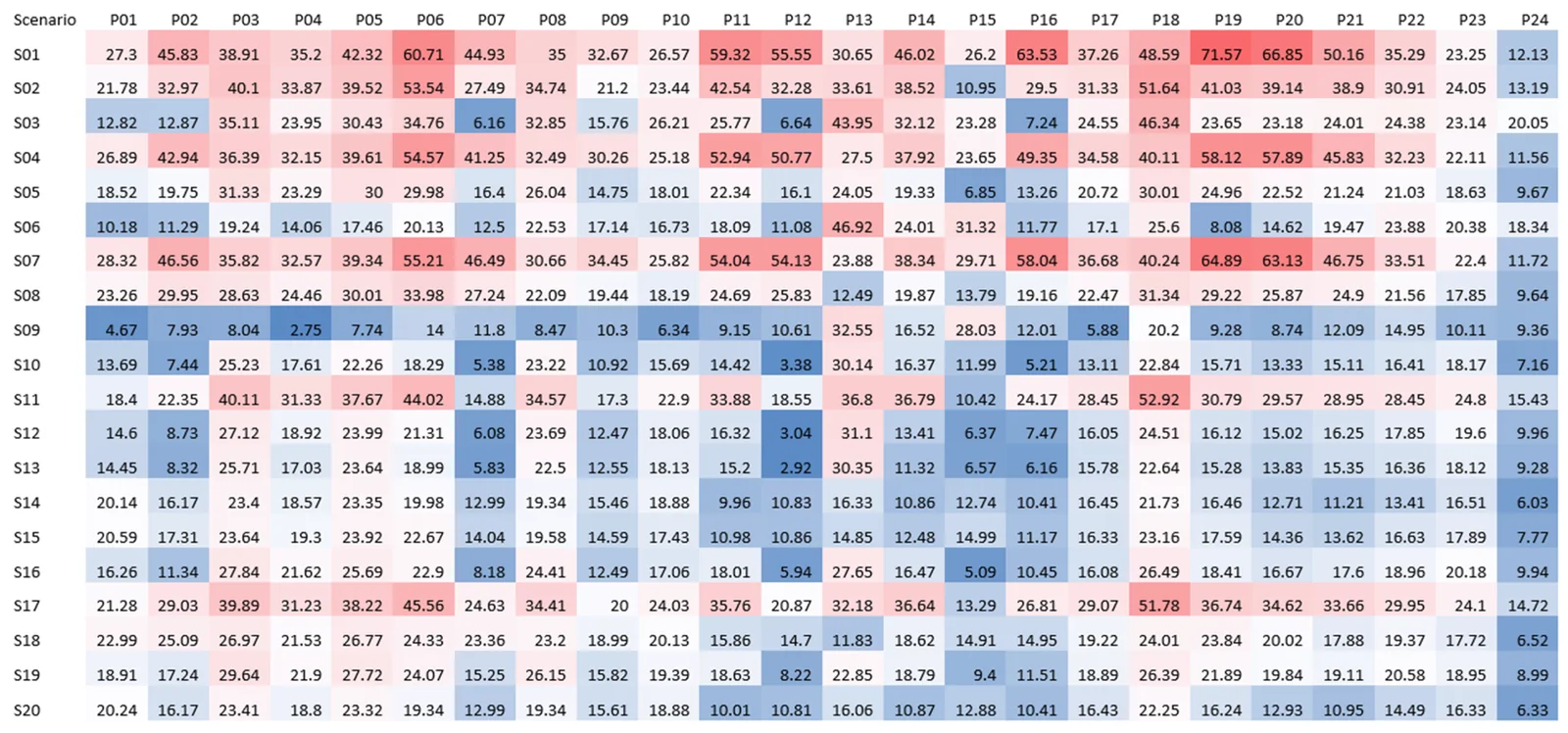
Heatmap of patch-wise CIEDE2000 color differences (ΔE_00_) obtained for the 20 experimental lighting scenarios and the 24 ColorChecker patches. Lower values, shown in blue shades, indicate smaller colorimetric deviations, whereas higher values, shown in red shades, indicate larger deviations.

To examine the combined influence of lighting scenario and target color, the patch-wise values obtained for all 20 scenarios were visualized as a heatmap (Figure 8). The resulting pattern reveals a pronounced dependence of colorimetric error on both illumination condition and ColorChecker patch. Scenarios characterized by low mean error, particularly S09 and S10, contain predominantly lower ΔE_00_ values, whereas the low-brightness white-light scenarios S01, S04, and S07 exhibit broader regions of elevated error. This confirms the general trends observed in Figure 6 while additionally showing that the magnitude of improvement is strongly color-dependent.

Certain patches were consistently more sensitive to changes in illumination. Across the complete set of scenarios, the Cyan patch (P18) and Bluish Green patch (P06) exhibited the highest average color differences, with mean ΔE_00_ values of approximately 32.6 and 31.9, respectively. Blue Sky (P03), Blue Flower (P05), and Blue (P13) also showed comparatively high deviations. In contrast, the Black patch (P24) exhibited the lowest overall mean error, followed by Red (P15). These results indicate that spectral changes in the illumination do not affect all target colors equally, with blue-green regions showing particularly pronounced sensitivity under the investigated conditions.

Importantly, no single lighting scenario minimized the color difference for all 24 patches. Although S09 provided the lowest overall mean ΔE_00_, the minimum error for individual patches was distributed among several scenarios, including S06, S09, S10, S13, S14, S16, S18 and S20.

**Figure 9:**
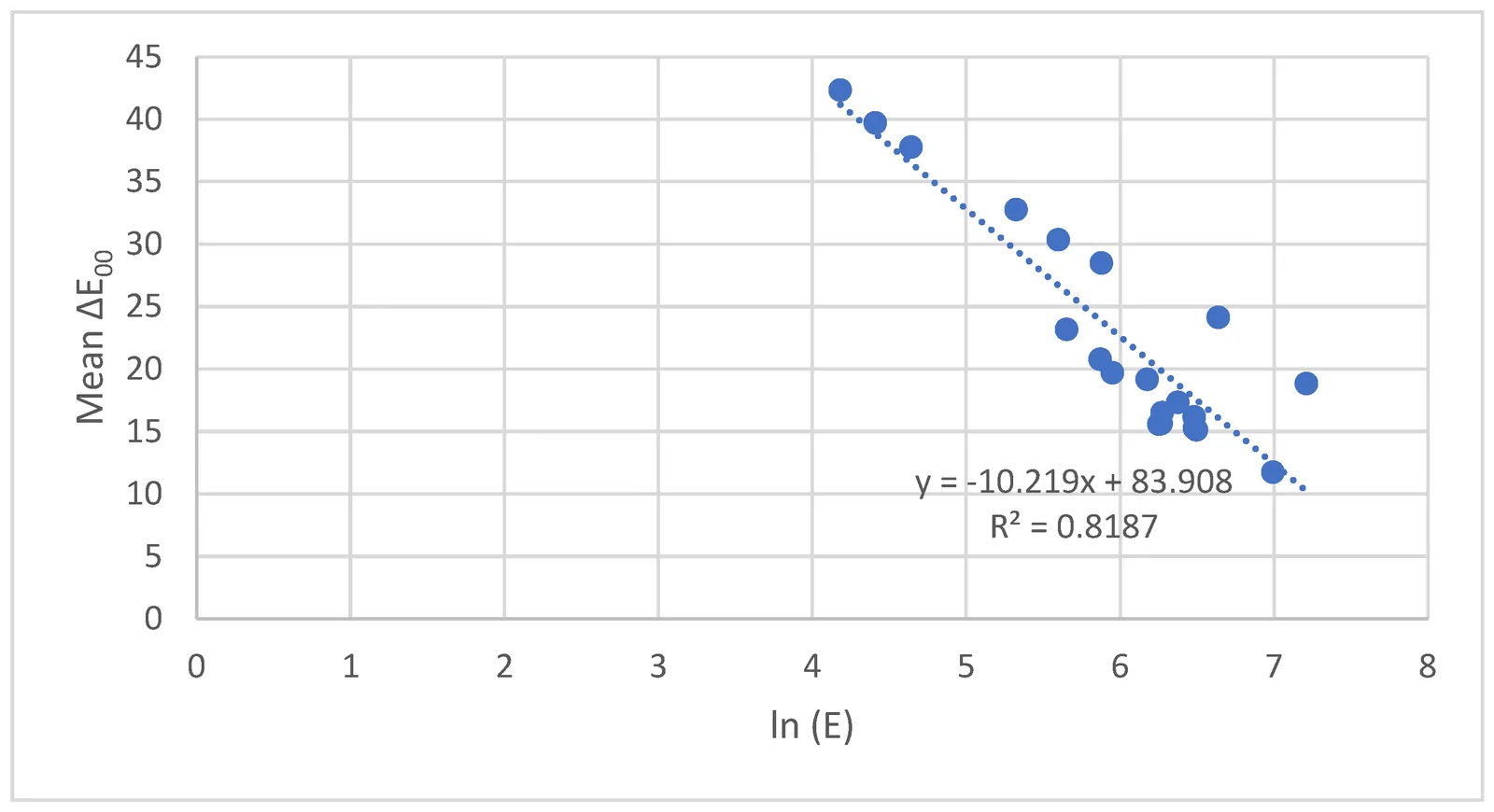
Relationship between mean CIEDE2000 color difference (ΔE_00_) and the natural logarithm of target-plane illuminance, ln(E), across the 20 experimental lighting scenarios. The dashed line represents the linear regression fit.

A strong inverse relationship was observed between mean colorimetric error and the logarithm of target-plane illuminance (Figure 9). Linear regression yielded ΔE_00_ = −10.219 ln(E) + 83.908, with R^2^ = 0.819, indicating that approximately 82% of the observed variation in mean ΔE_00_ was associated with the logarithmic variation in illuminance. The distribution of individual scenarios around the regression line nevertheless indicates that illuminance alone does not fully account for the observed colorimetric performance.

**Figure 10:**
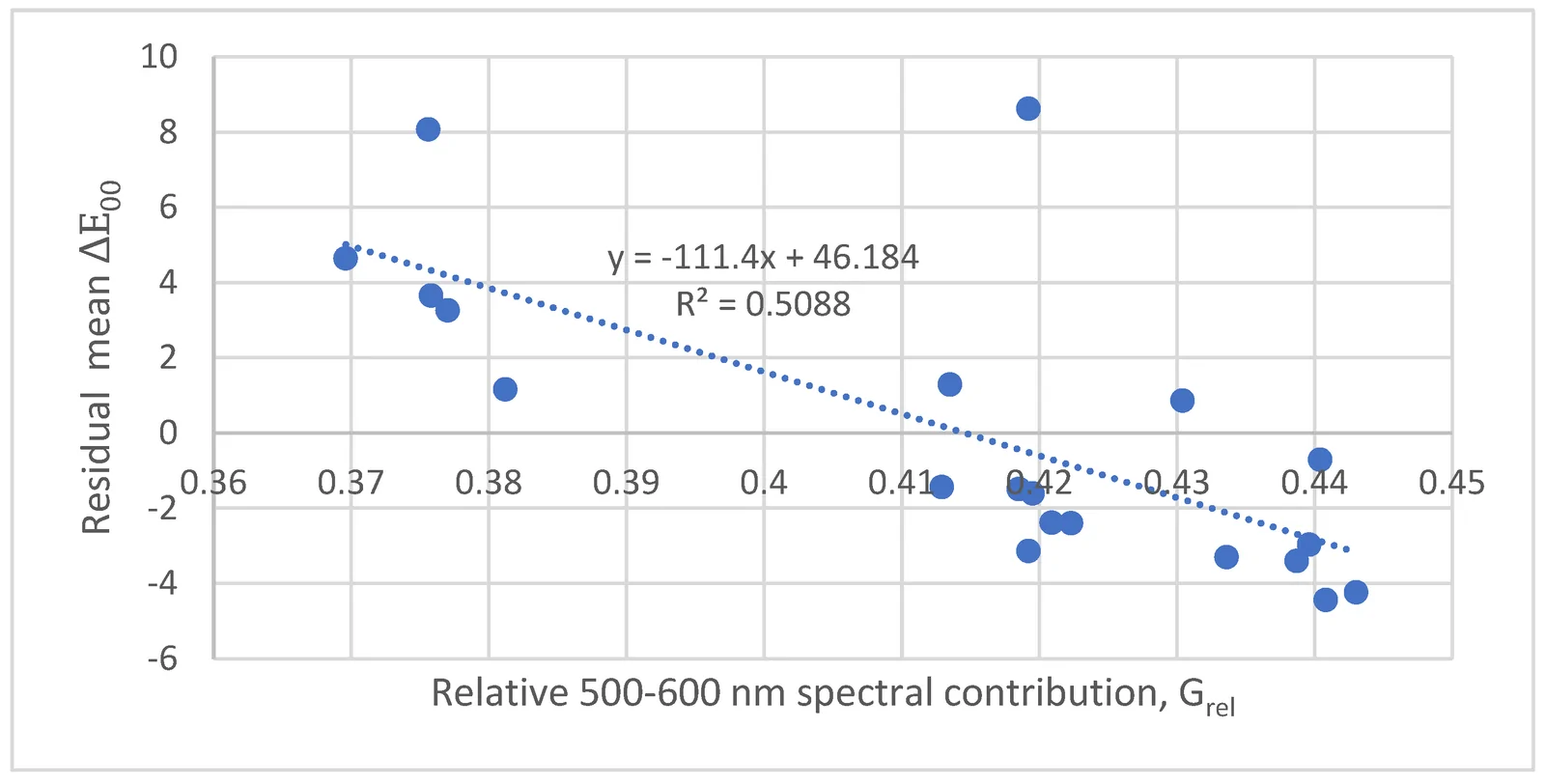
Relationship between residual mean CIEDE2000 color difference (ΔE_00_) and the relative spectral contribution in the 500–600 nm band (*G_rel_*) after accounting for the effect of illuminance. The dashed line represents the linear regression fit.

After accounting for the effect of illuminance using the logarithmic model presented in Figure 9, the residual mean colorimetric error showed a significant inverse relationship with *G_rel_*, the relative spectral contribution in the 500–600 nm band (Figure 10). Linear regression yielded R^2^ = 0.509, with a Pearson correlation coefficient of r = −0.713 (*p* < 0.001). Thus, scenarios characterized by a larger relative spectral contribution within the 500–600 nm range generally exhibited lower residual ΔE_00_ values.

The inclusion of the spectral descriptor *G_rel_* substantially improved the regression model compared with illuminance alone (Table 5). The coefficient of determination increased from R^2^ = 0.819 for the illuminance-only model to R^2^ = 0.919 when the relative 500–600 nm spectral contribution was included. The corresponding adjusted R^2^ increased from 0.809 to 0.909.

### 3.3. JPEG and RAW Saturation Behavior

A comparison between the simultaneously acquired JPEG and DNG images revealed differences in saturation behavior under the highest-output lighting conditions (Table 6). JPEG clipping was observed primarily in S03, S06, and S09, with mean clipped-pixel fractions across the 24 patches of 8.36%, 19.44%, and 1.27%, respectively. S06 showed the strongest JPEG saturation, with one patch reaching 99.59% clipped pixels. In contrast, the corresponding RAW data showed virtually no saturation; the maximum RAW clipping observed across the complete dataset was only 0.167% for an individual patch in S06. S10 exhibited only negligible JPEG clipping (0.02% mean; 0.40% maximum), while no clipping was detected in the remaining scenarios.

An additional repeatability experiment showed very low variability among the three separate image acquisitions performed for each lighting scenario. The standard deviation of the scenario-level mean ΔE_00_ values averaged 0.033 and did not exceed 0.115 across the 20 scenarios (Table 7). The corresponding coefficients of variation ranged from 0.007% to 0.582%, with a median value of 0.133%. Moreover, the mean values obtained from the three repeated acquisitions were also highly consistent with the original scenario-level results (Pearson r = 0.9988, *p* < 0.001; Spearman ρ = 0.9970, *p* < 0.001), with an RMSE of 0.502 ΔE_00_. 19 of the 20 scenarios differed from the original measurements by less than 1 ΔE_00_. These results indicate that image-acquisition variability was small compared with the differences observed among illumination scenarios.

## 4. Discussion

The results of this study indicate that illumination influences smartphone-based colorimetric readout through two coupled factors: the amount of incident light and its spectral composition. Increasing illumination generally reduced the colorimetric error under fixed smartphone acquisition conditions, as shown by the strong inverse relationship between mean ΔE_00_ and ln(E), for which the illuminance-only model reached R^2^ = 0.819. However, the dispersion of individual scenarios around this relationship shows that illuminance alone cannot fully explain the observed colorimetric response. This distinction is important because illuminance is weighted according to human photopic sensitivity and does not uniquely describe the spectral stimulus reaching the smartphone camera sensor. Consequently, two lighting conditions characterized by similar illuminance may produce different camera responses when their spectral power distributions differ [12].

This behavior is particularly evident in the direct comparison between S05 and S11. The two scenarios produced almost identical target-plane illuminance, 354.33 lx and 357.15 lx, respectively, corresponding to a difference of less than 1%, while their mean ΔE_00_ values were 20.78 and 28.48. Thus, the colorimetric error increased by approximately 37% despite the close photometric match. Their normalized spectral distributions were nevertheless markedly different, and the effect was strongly patch-dependent, with particularly large deviations for several blue-green patches. This comparison provides direct experimental evidence that photometrically similar illumination conditions do not necessarily provide equivalent conditions for smartphone colorimetric readout. Similar effects have been reported in camera-based colorimetry, where the interaction among illumination spectrum, target reflectance, and camera spectral sensitivity influences the resulting RGB signal [13]. Recent smartphone-colorimetry studies likewise report pronounced sensitivity to illumination conditions and show that calibration or color-space selection can substantially affect measurement robustness [20,21].

The spectral measurements further show that nominal brightness and apparent light color cannot be regarded as substitutes for spectral characterization. S17–S19 used the same unequal brightness distribution of 25%, 50%, and 75%, yet their normalized spectra remained clearly different because of their warm-, cool-, and neutral-white chromatic settings. Likewise, the mixed-white condition S10 approached but did not reproduce the spectral profile of S19. The color-accented scenarios S11–S16 also demonstrate that introducing a colored source into the multi-source system does not merely add an isolated narrow spectral component, but redistributes the overall spectral balance. Therefore, visually similar or nominally comparable lighting conditions may expose the colorimetric target to substantially different wavelength distributions. This is particularly relevant for camera-based biosensor readout because the recorded signal depends on the complete optical chain rather than on nominal lighting labels alone [13,24]. Moreover, the three-channel RGB representation does not uniquely preserve the spectral information of either the illumination or the target, since spectrally different illuminants may produce equivalent or closely related projections in the camera response space [24].

Spectral tuning was also associated with substantial differences in electrical power demand. Identical nominal brightness settings did not result in identical power consumption for different chromatic configurations. At 100% brightness, for example, the warm-, neutral-, and cool-white conditions S03, S06, and S09 consumed 15 W, 26 W, and 20 W, respectively. Similar differences were observed at lower brightness levels. Thus, spectral selection in a programmable multi-channel LED system affects not only the optical output and colorimetric response but also the electrical cost required to produce that illumination. The S05–S11 pair illustrates this trade-off particularly well: the two conditions produced virtually the same target-plane illuminance, whereas their electrical powers were 7 W and 6 W, corresponding to target-plane illuminance-to-power ratios of 50.62 and 59.53 lx/W. Nevertheless, the more favorable E/P ratio of S11 was accompanied by substantially poorer colorimetric performance. Consequently, maximizing photometric output per electrical watt does not necessarily minimize colorimetric error. These observations suggest that illumination selection for smartphone colorimetric readout can be considered as a multi-objective problem involving spectral suitability, colorimetric accuracy, and electrical power demand.

The patch-wise results further indicate that the effect of illumination is strongly color-dependent. Several blue-green patches exhibited comparatively large deviations, and no single lighting scenario minimized ΔE_00_ for all 24 ColorChecker patches. Although S09 produced the lowest overall mean color difference, the minimum patch-specific errors were distributed among several scenarios. This finding argues against the assumption that a single universally optimal illumination condition exists for all colorimetric targets. Instead, the preferred spectral distribution may depend on the spectral characteristics of the particular chromogenic marker or reaction product being quantified. For smartphone-based biosensors, illumination optimization should therefore be regarded as potentially marker-dependent rather than simply as the identification of a single “best” white-light condition. This interpretation is consistent with the diversity of chromogenic assays and controlled-illumination approaches reported in smartphone-based biochemical sensing [1].

The regression analysis provides quantitative support for this interpretation. After the influence of illuminance was accounted for, the residual colorimetric error remained significantly associated with G_rel_, defined as the relative spectral contribution in the 500–600 nm band. While the illuminance-only model explained 81.9% of the variation in mean ΔE_00_, inclusion of the spectral descriptor increased R^2^ to 0.919 and the adjusted R^2^ from 0.809 to 0.909. The spectral term therefore contributed information that was not contained in illuminance alone. The investigated 500–600 nm interval encompasses the region around the maximum of the photopic visual sensitivity function; however, this result should be interpreted as an empirical descriptor of the present smartphone-illumination-target configuration rather than as evidence that this band is universally optimal for colorimetric biosensing. Smartphone camera spectral sensitivities differ from the human V(λ) function and can vary substantially among devices [14]. Cross-device differences in smartphone colorimetry have also been demonstrated experimentally under controlled color-reference conditions [19]. Validation across additional camera sensors and chromogenic targets will therefore be required before generalizing the identified spectral interval.

The comparison between simultaneously acquired JPEG and RAW images highlights a second practical limitation of increasing illumination. Under the highest-output conditions, clipping was substantially more pronounced in the processed JPEG images than in the corresponding RAW data. In S06, the mean JPEG clipped-pixel fraction reached 19.44%, while an individual ColorChecker patch reached 99.59% clipping; in contrast, RAW clipping remained negligible. Therefore, increasing illumination can improve colorimetric performance at low and moderate light levels, but excessive illumination may drive the processed JPEG representation into saturation and remove color information that may still be retained in the corresponding RAW data. This distinction is particularly relevant for quantitative smartphone imaging because JPEG generation involves several internal processing stages, including interpolation, color-channel adjustment, and compression, whereas RAW data retain a representation closer to the original sensor response [2,15]. Moreover, when a weakly colored assay is difficult to interpret visually, a user may naturally attempt to place it under stronger illumination to make the marker more visible. The present results indicate that such an intuitive strategy may become counterproductive once the processed image reaches saturation.

These observations support an adaptive illumination strategy in which both spectral composition and intensity are selected according to the expected colorimetric response while exposure is maintained below the saturation region of the smartphone imaging pipeline. In such a configuration, programmable LED illumination becomes an active component of the optical readout system rather than merely a means of increasing scene brightness. The illumination spectrum could therefore be adjusted according to the relevant chromogenic marker, while electrical power demand and the risk of JPEG saturation are simultaneously considered. Multi-objective spectral optimization has previously been applied to tunable LED systems to balance color quality, luminous efficacy and energy-related performance [22,23]. The present results extend this concept to smartphone-based colorimetric readout by jointly considering illumination spectrum, colorimetric error, electrical power demand and image-saturation constraints.

The repeatability assessment supports the stability of the experimental methodology at both the illumination and image-acquisition levels. The repeated lighting scenarios S14 and S20 showed highly similar spectral characteristics, while the three repeated smartphone acquisitions performed for each of the 20 lighting scenarios exhibited very low within-scenario variability, with a median coefficient of variation of 0.133%. The repeated-acquisition means also closely agreed with the original measurements (Pearson r = 0.9988). These findings indicate that image-acquisition variability was small relative to the differences observed among illumination scenarios. Nevertheless, the scope of the present study imposes several limitations that should be considered when interpreting these results.

First, the optical readout methodology was evaluated using a standardized ColorChecker reference rather than a specific biological assay. The ColorChecker was selected to provide a controlled and reproducible optical reference for isolating the illumination-camera interaction, but it should not be regarded as a surrogate for validation of a specific biosensor assay. Future work should therefore evaluate the proposed illumination-selection methodology using representative chromogenic biosensors covering different color-development mechanisms and dynamic ranges.

Second, only one smartphone model was evaluated. Because smartphone cameras may differ substantially in spectral sensitivity, optics, and internal image-processing pipelines, the quantitative relationships identified in this study, including the contribution of the 500–600 nm spectral descriptor, should be regarded as specific to the investigated camera-illumination configuration rather than as device-independent relationships. Cross-device studies using smartphones with different camera characteristics will therefore be necessary to assess the generalizability of the proposed methodology.

The findings should therefore be interpreted as validation of the illumination-selection and smartphone colorimetric-readout methodology, rather than as validation of a specific biosensor. Future work should extend the approach to representative colorimetric biosensors, different chromogenic response ranges, additional smartphone cameras, and less controlled acquisition conditions more representative of routine end-user operation.

Overall, the results indicate that reliable smartphone colorimetric readout requires illumination to be treated as a spectrally defined measurement parameter with an associated energetic and dynamic-range cost, rather than merely as a means of increasing scene brightness.

## 5. Conclusions

This study demonstrates that smartphone-based colorimetric readout is governed not only by illumination level but also by the spectral composition of the incident light. Across the 20 programmable LED scenarios, mean ΔE00 varied from 11.73 to 42.33. Two conditions differing by less than 1% in illuminance produced approximately 37% different mean colorimetric errors, confirming that photometrically similar lighting conditions are not necessarily equivalent for camera-based colorimetry. The illuminance-only model accounted for 81.9% of the variation in mean ΔE_00_, while the addition of the relative spectral descriptor increased the explained variance to 91.9%.

The repeatability assessment showed very low image-acquisition variability across all 20 lighting scenarios, while the repeated S14/S20 lighting condition also showed close spectral agreement, supporting the stability of the experimental methodology. At the same time, the patch-wise analysis showed that no single illumination scenario minimized colorimetric error for all target colors, indicating that the optimum spectral condition may depend on the specific chromogenic marker being evaluated.

The results further show that illumination optimization should also consider electrical power demand and the dynamic range of the smartphone imaging pipeline. High-output conditions produced substantial JPEG clipping while RAW saturation remained negligible. Overall, programmable illumination should be treated as an active and spectrally defined component of the smartphone colorimetric measurement chain. The proposed spectral-colorimetric-energetic perspective provides a basis for future optimization with real chromogenic biosensors and additional smartphone platforms.

## Figures and Tables

**Table 1:** Geometric parameters of the experimental setup.

Component	Distance to Target Center [cm]	Height [cm]	Azimuth [°]	Tilt [°]
LED1	65	15	−30	30
LED2	76	15	0	45
LED3	65	15	+30	30
Smartphone camera	76	22.8	0	0

**Table 2:** Experimental Lighting Scenarios.

Scenario	LED 1—Color	Brightness	LED 2—Color	Brightness	LED 3—Color	Brightness	Purpose/Spectral Characteristic
S01	Warm white	25%	Warm white	25%	Warm white	25%	Warm white—low level
S02	Warm white	50%	Warm white	50%	Warm white	50%	Warm white—medium level
S03	Warm white	100%	Warm white	100%	Warm white	100%	Warm white—high level
S04	Neutral white	25%	Neutral white	25%	Neutral white	25%	Neutral white—low level
S05	Neutral white	50%	Neutral white	50%	Neutral white	50%	Neutral white—medium level
S06	Neutral white	100%	Neutral white	100%	Neutral white	100%	Neutral white—high level
S07	Cool white	25%	Cool white	25%	Cool white	25%	Cool white—low level
S08	Cool white	50%	Cool white	50%	Cool white	50%	Cool white—medium level
S09	Cool white	100%	Cool white	100%	Cool white	100%	Cool white—high level
S10	Warm white	75%	Neutral white	75%	Cool white	75%	Mixed white illumination
S11	Red	50%	Warm white	75%	Warm white	75%	Predominant R spectral contribution
S12	Yellow	50%	Neutral white	75%	Neutral white	75%	Combined R + G spectral contribution
S13	Green	50%	Neutral white	75%	Neutral white	75%	Predominant G spectral contribution
S14	Cyan/Light blue	50%	Cool white	75%	Cool white	75%	Combined G + B spectral contribution
S15	Blue	50%	Cool white	75%	Cool white	75%	Predominant B spectral contribution
S16	Magenta/Pink	50%	Neutral white	75%	Neutral white	75%	Combined R + B spectral contribution
S17	Warm white	25%	Warm white	50%	Warm white	75%	Warm-white illumination with unequal source intensities
S18	Cool white	25%	Cool white	50%	Cool white	75%	Cool-white illumination with unequal source intensities
S19	Neutral white	25%	Neutral white	50%	Neutral white	75%	Neutral white illumination with unequal source intensities
S20	Cyan/Light blue	50%	Cool white	75%	Cool white	75%	Repeat of S14 for repeatability assessment

**Table 3:** Measured photometric, radiometric, colorimetric, and electrical parameters of the experimental lighting scenarios.

Scenario	E [lx]	Ee [W/m^2^]	CCT [K]	CRI (Ra)	x	y	P [W]	E/P [lx/W]
S01	65.5	0.223	3051	90.5	0.4289	0.3959	1 ± 0.5	65.5
S02	205.25	0.691	2704	90.0	0.4562	0.4047	4 ± 0.0	51.31
S03	764.26	2.564	2607	89.4	0.4649	0.4076	15 ± 0.0	50.95
S04	103.72	0.345	4152	93.2	0.3747	0.3755	2 ± 0.3	51.86
S05	354.33	1.158	3948	93.7	0.3836	0.3810	7 ± 0.0	50.62
S06	1355.25	4.414	3861	94	0.3873	0.3825	26 ± 0.0	52.13
S07	82.28	0.271	5405	88.1	0.3348	0.3526	1 ± 0.5	82.28
S08	285.31	0.922	5314	88.3	0.3372	0.3540	4 ± 0.0	71.33
S09	1089.92	3.515	5338	89.0	0.3365	0.3531	20 ± 0.0	54.5
S10	662.97	2.164	4113	92.8	0.3755	0.3734	12 ± 0.0	55.25
S11	357.15	1.207	2576	90.3	0.4656	0.4047	6 ± 0.0	59.53
S12	653.54	2.127	3804	94.6	0.3900	0.3839	10 ± 0.0	65.35
S13	654.12	2.124	3876	94.3	0.3875	0.3850	10 ± 0.4	65.41
S14	519.24	1.680	5426	89.1	0.3343	0.3535	8 ± 0.0	64.91
S15	530.83	1.738	5541	89.8	0.3315	0.3476	8 ± 0.0	66.35
S16	587.20	1.929	3819	95.1	0.3380	0.3794	10 ± 0.0	58.72
S17	270.06	0.907	2622	89.6	0.4640	0.4079	5 ± 0.0	54.01
S18	383.92	1.243	5323	88.7	0.3369	0.3531	6 ± 0.0	63.99
S19	481.71	1.569	3849	93.7	0.3879	0.3829	8 ± 0.0	60.21
S20	526.66	1.697	5405	88.8	0.3349	0.3546	8 ± 0.0	65.83

**Table 4:** Colorimetric performance of the smartphone JPEG images under the 20 experimental lighting scenarios.

Scenario	Mean ΔE_00_	SD	Median	Max ΔE_00_
S01	42.33	15.14	40.62	71.57
S02	32.76	10.40	33.29	53.54
S03	24.13	10.56	23.98	46.34
S04	37.76	12.19	37.16	58.12
S05	20.78	6.28	20.88	31.33
S06	18.83	8.11	17.78	46.92
S07	39.70	13.67	37.51	64.90
S08	23.16	6.13	23.86	33.98
S09	11.73	6.89	9.73	32.55
S10	15.13	6.67	15.40	30.14
S11	28.48	10.18	28.70	52.92
S12	16.17	7.06	16.19	31.10
S13	15.26	6.78	15.32	30.35
S14	15.58	4.56	16.25	23.40
S15	16.49	4.36	16.48	23.92
S16	17.32	6.63	17.33	27.84
S17	30.35	9.20	30.59	51.79
S18	19.70	4.93	19.69	26.97
S19	19.17	5.70	19.03	29.64
S20	15.63	4.53	16.11	23.41

**Table 5:** Comparison of regression models for predicting mean colorimetric error.

Model	Predictors	Regression Equation	R^2^	Adjusted R^2^
M1	Illuminance	ΔE_00_ = 83.908–10.219 ln(E)	0.819	0.809
M2	Illuminance + spectral contribution	ΔE_00_ = 127.82–9.16 ln(E) − 121.06 G_rel_	0.919	0.909

**Table 6:** JPEG and RAW clipping under high-output lighting conditions.

Scenario	Mean JPEG Clipped Pixels [%]	Maximum JPEG Clipping in a Patch [%]	Mean RAW Clipped Pixels [%]	Maximum RAW Clipping in a Patch [%]
S03	8.36	72.39	0.000	0.000
S06	19.44	99.59	0.007	0.167
S09	1.27	30.31	0.000	0.000
S10	0.02	0.40	0.000	0.000

**Table 7:** Repeatability of smartphone image acquisition across the 20 lighting scenarios.

Scenario	Mean ΔE_00_, Repeated Acquisitions	SD	CV [%]
S01	42.72	0.065	0.15
S02	32.72	0.041	0.13
S03	24.27	0.014	0.06
S04	38.00	0.003	0.01
S05	22.50	0.005	0.02
S06	19.50	0.022	0.11
S07	40.12	0.028	0.07
S08	22.71	0.017	0.08
S09	12.51	0.052	0.41
S10	15.37	0.035	0.23
S11	28.47	0.015	0.05
S12	16.22	0.028	0.17
S13	15.28	0.023	0.15
S14	15.68	0.027	0.17
S15	16.46	0.046	0.28
S16	17.23	0.087	0.50
S17	30.33	0.012	0.04
S18	19.72	0.028	0.14
S19	19.72	0.115	0.58
S20	15.67	0.005	0.03

Values represent the mean, standard deviation (SD), and coefficient of variation (CV) of the scenario-level mean ΔE_00_ obtained from three separate smartphone image acquisitions per lighting scenario.

## Data Availability

The datasets generated and analyzed during the current study are available from the corresponding author upon reasonable request.
